# A Rare Case of Collecting Duct Carcinoma With Extensive Coagulative Necrosis

**DOI:** 10.7759/cureus.49295

**Published:** 2023-11-23

**Authors:** Robert Colef, Leslie Mescallado, Nfn Kiran, Monika Wrzolek, Shahbaz Khan

**Affiliations:** 1 Pathology and Laboratory Medicine, Northwell Health, New York City, USA; 2 Pathology and Laboratory Medicine, Staten Island University Hospital, New York City, USA; 3 Gastrointestinal, Hepatobiliary, and Transplant Pathology, Indiana University School of Medicine, Indianapolis, USA; 4 Hematopathology, Northwell Health, New York City, USA; 5 Pathology, University of Oklahoma Health Sciences Center, Oklahoma City, USA

**Keywords:** rare renal malignancy, extensive necrosis, bellini carcinoma, metastatic renal medullary, collecting duct carcinoma

## Abstract

Collecting duct carcinoma (CDC) is an aggressive renal malignancy with limited diagnostic and therapeutic consensus. We report a case of a 69-year-old male with CDC and extensive coagulative necrosis who presented with lower extremity swelling, abdominal distention, and an enlarged left kidney causing grade IV hydronephrosis. Initial treatment with a left percutaneous nephrostomy was followed by clinical deterioration and a diagnosis of emphysematous pyelonephritis. Pathological examination of drainage material revealed extensive coagulative necrosis and was suggestive of a necrotic neoplasm. Subsequent left nephrectomy confirmed CDC with high-grade features, stromal desmoplasia, and extensive coagulative necrosis. Immunohistochemistry studies supported the diagnosis. This study highlights the diagnostic complexity of CDC and emphasizes the need for accurate reporting of atypical presentations. CDC remains a formidable clinical entity with limited treatment options and poor outcomes. Further research is essential to enhance our understanding and management of this rare and aggressive renal malignancy.

## Introduction

Collecting duct carcinoma (CDC), identified as a distinct variant of renal cell carcinoma (RCC) by Fleming and Lewi in 1986, is a rare, highly aggressive carcinoma of the renal medulla, arising from the principal cells of the distal collecting ducts of Bellini, with a poor prognosis and an incidence of less than 1% of all RCCs [1-5]. CDC has a male-to-female ratio of 2:1, with the typical age of presentation occurring during the sixth decade of life [1]. CDC tumors commonly manifest with irregular infiltrative growth patterns, absent of extensive hemorrhage and necrosis [3,6]. Angulated tubules, tubulopapillary structures, and glandular formations are the predominant architectural configurations seen in CDC [3]. High-grade nuclear attributes, characterized by pleomorphism and conspicuous eosinophilic nucleoli, are frequently observed [7]. While focal necrosis may occasionally manifest as part of CDC's presentation, extensive necrosis remains an unusual presentation with a single documented case reported by Xu et al. [8]. For patients diagnosed with localized CDC, the standard treatment approach is nephrectomy [9].

## Case presentation

A 69-year-old male with a history of hiatal hernia, iron deficiency anemia, and untreated *Helicobacter pylori* infection presented to the emergency department (ED) with lower extremity swelling, abdominal distention, intermittent left lower quadrant pain, generalized weakness, and dyspnea on ambulation. Vital signs at presentation were relatively stable, and laboratory findings included a white blood cell count of 15 K/µL, hemoglobin level of 9.4 g/dL, platelet count of 505 µL, and creatinine level of 1 mg/dL. Urinalysis results were negative. Contrast-enhanced CT imaging revealed grade IV hydronephrosis of the left kidney, kidney enlargement with the kidney measuring 26 × 26 × 21 cm, and minimal residual renal parenchyma. The enlarged left kidney was noted to displace retroperitoneal structures to the right side and compress the inferior vena cava. Left percutaneous nephrostomy (PCN) placement was subsequently performed for renal decompression, and urine cytology was noted to be negative.

The patient's condition deteriorated with chief complaints of worsening weakness, abdominal distension, and no output from his left PCN. The PCN, which initially drained serosanguinous fluid, had changed to brown liquid output. Additionally, the patient was noted to have new-onset atrial fibrillation with a rapid ventricular response exceeding 150 beats per minute and was experiencing difficulty breathing, leading to the initiation of bilevel-positive airway pressure (BiPAP) therapy. Initial laboratory findings showed a white blood cell count of 47 K/µL, a serum creatinine level of 2.9 mg/dL, and a lactate level of 6 mmol/L. A CT scan of the abdomen and pelvis with intravenous contrast revealed free intraperitoneal fluid and air from an unknown source and fluid and air in the left kidney. The patient was diagnosed with emphysematous pyelonephritis. A percutaneous drainage procedure was undertaken on a left kidney abscess cavity identified on imaging. Pathology findings from this procedure revealed fragments of extensively necrotic tissue, which could not be fully characterized due to a lack of viable material. Immunohistochemical studies were performed, but due to the necrotic nature of the sample, they were inconclusive. However, the AE1/AE3 stain showed sheets of positive necrotic cells, while the CK7 and CK20 stainings were focal and weak. The findings, in conjunction with the H&E morphology, suggested the possibility of a necrotic neoplasm. Limited neutrophilic inflammation was also observed and interpreted as a reaction to the necrotic tumor with a possible coexisting or superimposed infection.

**Figure 1:**
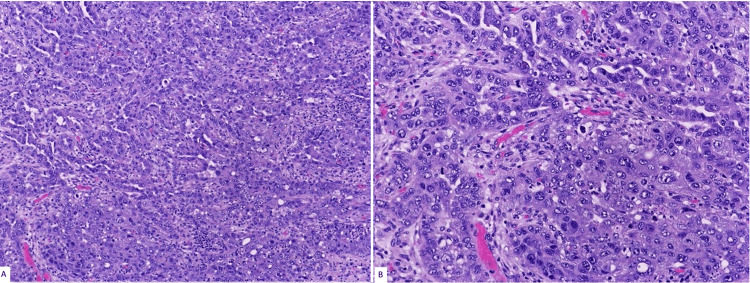
Microscopy of collecting duct carcinoma (hematoxylin and eosin stain {A: 100× and B: 200×}).

The patient subsequently underwent a left nephrectomy that grossly displayed a tan friable mass in the renal pelvis and medulla, with multiple cystic cavity areas and extensive necrotic material. On histology, collecting duct carcinoma was identified (Figures 1A, 1B).

**Figure 2:**
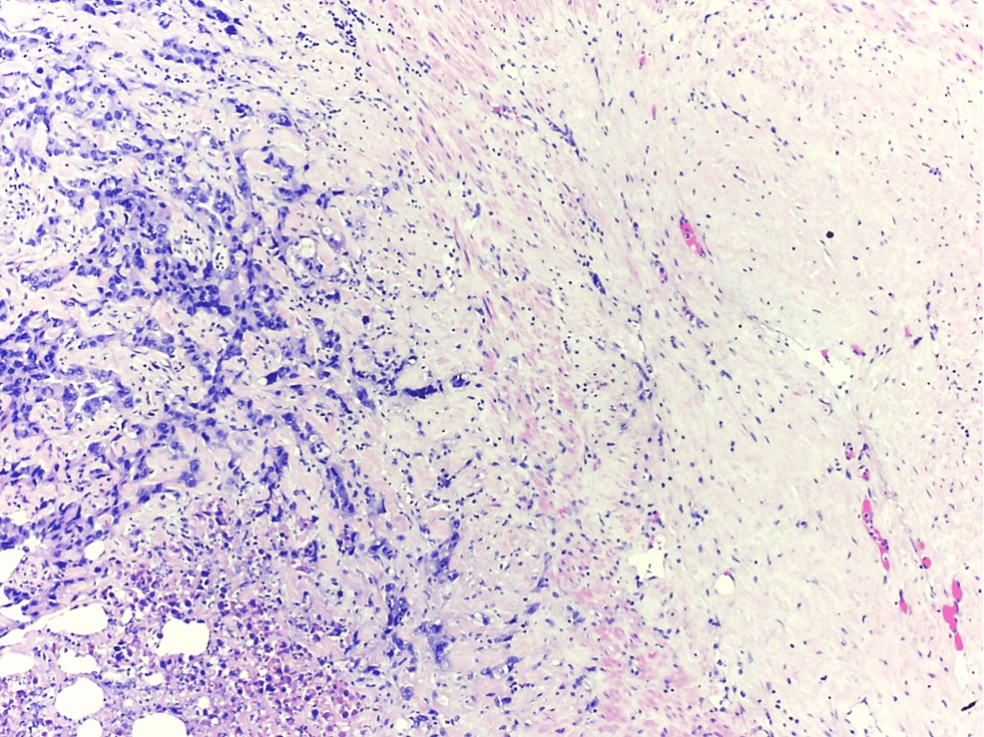
Microscopy of extensive necrosis (hematoxylin and eosin stain, 40×).

The carcinoma exhibited high-grade features of round to polygonal neoplastic cells with prominent nucleoli arranged in tubulopapillary and glandular structures with high nuclear grade, stromal desmoplasia, infiltrative growth, extensive coagulative necrosis, and abscess formation (Figure 2).

**Figure 3:**
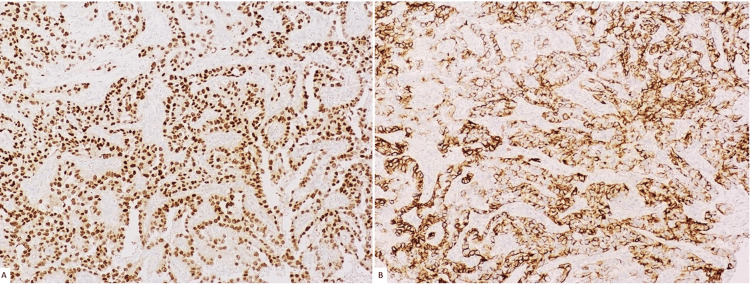
Immunohistochemical staining of neoplastic cells shows strong and diffuse (A) PAX8 stain (100×) and (B) CK7 stain (100×). PAX8: paired-box gene 8

Resection margins were positive, with tumor extension into the hilum and beyond Gerota's fascia through a perforation, thereby staging the tumor as pT4. Immunohistochemical staining showed strong and diffuse paired-box gene 8 (PAX8) and CK7 staining, with patchy and weak CD10 and GATA-3 staining, and negativity for RCC, HMWCK, p63, P504S, S100, CK20, SMA, and desmin (Figures 3A, 3B).

## Discussion

CDC is a rare, aggressive tumor with a poor prognosis that originates in the renal medulla and infiltrates the cortex and renal pelvis. Unfortunately, to date, the imaging features of CDC are not well characterized [10]. Due to the absence of specific imaging characteristics, identifying CDC based solely on imaging is exceptionally difficult. Consequently, an accurate diagnosis relies on pathological examination. The International Society of Urological Pathology established criteria for diagnosing CDC, which include the following pathological features: tumor involvement in the renal medulla, a predominant formation of tubules, the presence of a desmoplastic stromal reaction, the presence of high-grade cytologic features, an infiltrative growth pattern, and the exclusion of other subtypes of renal cell carcinoma or urothelial carcinoma [11]. Differential diagnosis of CDC includes urothelial carcinoma arising from the renal pelvis and SMARCB-1 deficient medullary-like renal carcinoma (SD-MLC). There is morphologic overlap between SD-MLC and CDC, with cribriform and reticular patterns favoring the former and papillary patterns favoring the latter, as seen in our patient. Immunohistochemical (IHC) staining can be used to differentiate between the two entities, HMWCK in CDC often stains positive and HMWCK stains negative in SD-MLC. Urothelial carcinoma has a predominantly solid architecture and is most commonly presented as a high-grade carcinoma, a shared feature with CDC. A careful examination of IHC stains is necessary to differentiate between CDC and urothelial carcinomas since they share similar IHC features. Paired-box gene 8 (PAX8) and p63 have emerged as important IHC markers in that effort with CDC showing positivity for PAX8, while p63 is positive in urothelial carcinoma cases. Therefore, PAX8+/p63- strongly favors CDC, while PAX8-/p63+ favors urothelial carcinoma [12].

CDC cases are characterized by their high-grade and aggressive nature, yet there is currently no consensus regarding the optimal treatment approach. Surgery remains the most viable option, demonstrating effectiveness even in advanced cases with radical nephrectomy being the recommended choice due to the invasiveness of CDC, often representing the only potentially curative option for CDC patients [13-15]. Survival outcomes are significantly influenced by treatment, as demonstrated by Sui et al. who found that surgery or surgery combined with chemotherapy and/or radiation conferred a survival advantage over no treatment, chemotherapy and/or radiation therapy alone did not exhibit improved survival compared to no treatment, and the addition of chemotherapy and/or radiation to surgery did not yield additional benefits over surgery alone in their subgroup analysis [16]. Despite surgical intervention, the prognosis for CDC remains bleak, with a median survival time of 13 months after diagnosis [16]. Even with the incorporation of chemotherapy and targeted therapy, there has been no substantial improvement in survival rates, indicating that adjuvant therapies have not yielded satisfactory results in enhancing patient survival [17]. Collectively, these findings underscore the challenging nature of CDC management and the imperative need for more effective treatment strategies to improve patient outcomes.

## Conclusions

Collecting duct carcinoma is an aggressive renal neoplasm with a low incidence rate, poor prognosis, and characteristic histopathologic features. With no demonstrated effective adjuvant therapies, surgery remains the cornerstone of management. Given the unusual clinical presentation of an already rare malignancy, it is important to report cases of CDC with extensive coagulative necrosis to raise awareness and improve patient outcomes.
